# Mass‐Forming Variants in Antineutrophil Cytoplasmic Antibody–Associated Vasculitis: Diagnostic Complexities in Granulomatous Disease. A Case Report

**DOI:** 10.1002/acr2.70038

**Published:** 2025-04-04

**Authors:** Benedetta Fazzi, Elena Treppo, Simone Longhino, Maria Pillon, Luca Quartuccio

**Affiliations:** ^1^ University of Udine, Academic Hospital “Santa Maria della Misericordia,” Udine Italy

## Abstract

A middle‐aged woman presented with a granulomatous breast lesion in 2018. By 2021, antibiotic‐resistant pneumonia led to the discovery of granulomatous inflammation in the lung and thyroid. Initially misdiagnosed as Erdheim–Chester disease (ECD), she was treated with interferon without success. Histopathology later ruled out ECD, suggesting an unspecified granulomatous disease, with granulomatosis with polyangiitis (GPA) initially excluded due to negative antineutrophil cytoplasmic antibodies (ANCAs) and foamy histiocytes. In 2023, repeated lung biopsy and PR3‐ANCA positivity led to a revised diagnosis of mass‐forming GPA. Rituximab therapy resulted in remission. This case highlights the diagnostic complexity of GPA with atypical histopathological features.

## Introduction

Granulomatosis with polyangiitis (GPA) is a rare antineutrophil cytoplasmic antibody–associated vasculitis (AAV) characterized by necrotizing granulomatous inflammation, predominantly affecting the respiratory tract and kidneys. Mass‐forming variant of GPA is exceptionally rare and often misdiagnosed as malignancies or other granulomatous disorders. The presence of foamy histiocytes is an unusual feature in GPA, further complicating diagnosis. Currently, there are no established therapeutic recommendations specifically addressing the treatment of mass‐forming variants of GPA.^1^ The authors affirm that human participants provided informed consent for publication of the images. Written informed consent was obtained from all individual participants included in the study.

## Case report

**Figure 1:**
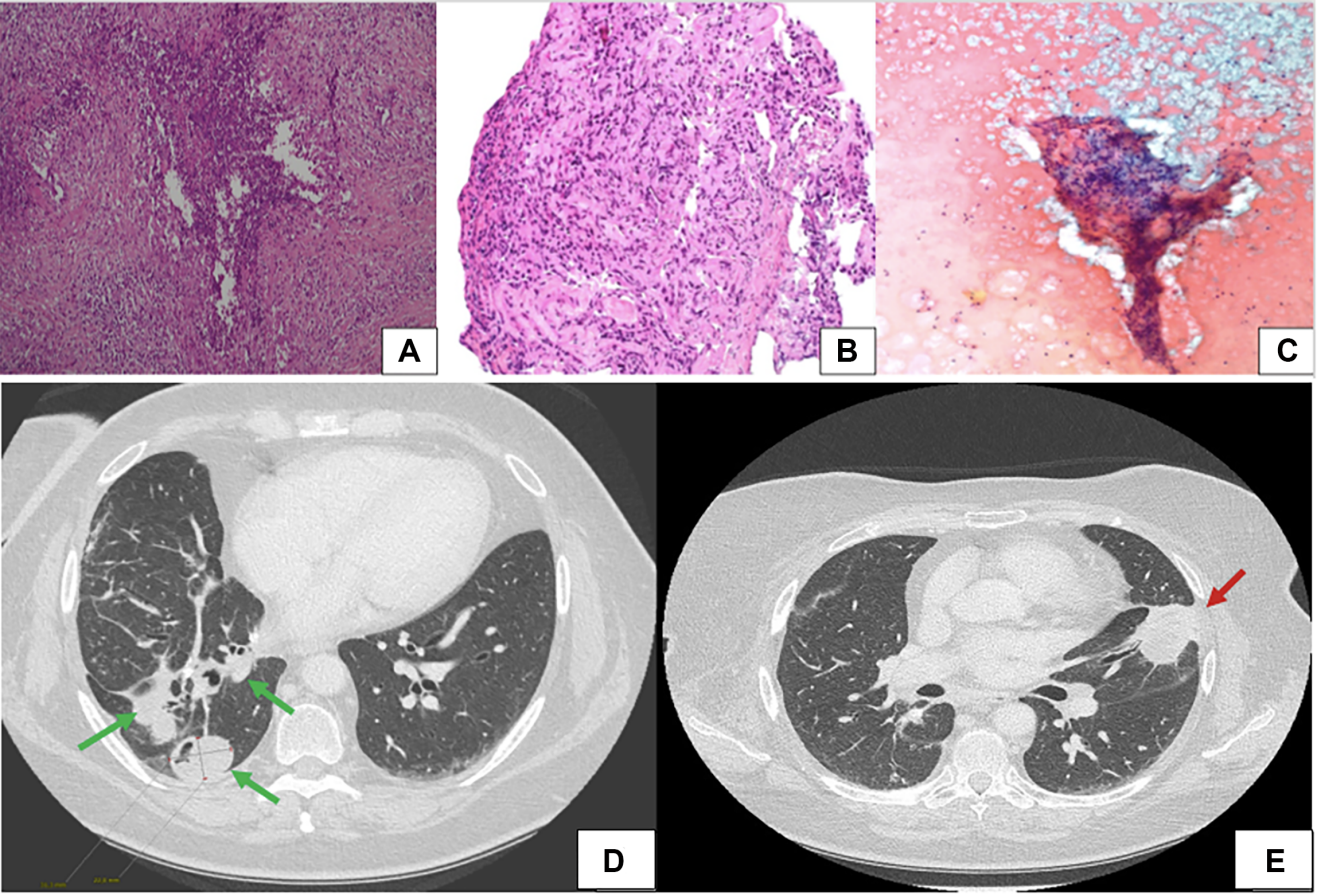
Histopathological micrographs are shown of the (A) left breast, central necrotizing granuloma surrounded by diffuse inflammation and foamy hystiocytes (magnification 10x); (B) right lung, chronic lymphoid inflammation and pneumocytic activation (magnification 20x); and (C) left lobe of the thyroid, histiocytic cluster with granulomatous feature in a carpet‐like arrangement of inflammatory granulocytes. Chest CT scans are shown of the (D) right lung, multiple calcified nodules and a newly developed nodule measuring approximately 3 cm in diameter (green arrows); and (E) left lung, lingular region, a pleura‐based consolidation measuring approximately 3.5 cm in diameter (red arrow). CT, computed tomography.

A middle‐aged woman underwent a routine mammogram in 2018, revealing a consolidation in the left breast. A quadrantectomy was performed, and histopathology showed inflammatory infiltrates with foamy histiocytes but no malignancy (Figure 1A). The patient remained asymptomatic until 2021, when she developed fever unresponsive to antibiotics. Imaging identified a basal consolidation in the right lung, and a surgical biopsy revealed granulomatous inflammation and foamy histiocytes (Figure 1B). A similar consolidation appeared in the left lung months later, leading to a bronchoscopy with transbronchial biopsy, confirming a similar histologic pattern. Laboratory tests showed significant inflammation (C‐reactive protein > 80 mg/L, fibrinogen > 800 mg/dL) but negative autoantibodies, including antineutrophil cytoplasmic antibodies (ANCAs).

The patient was initially treated with prednisone (50 mg/day), leading to temporary symptom relief. A concurrent thyroid ultrasound identified a 23‐mm hypoechoic nodule, and fine‐needle aspiration confirmed granulomatous inflammation with histiocytic clusters (Figure 1C). The presence of foamy histiocytes across multiple organs prompted consideration of Erdheim–Chester disease (ECD), supported by positive CD68 staining. However, the absence of the BRAF V600E mutation made her ineligible for targeted therapy. Instead, pegylated alpha‐interferon was initiated, but within a few months, the patient developed new symptoms, including polyarthralgia, persistent fever, and purpuric lesions. A skin biopsy showed leukocytoclastic vasculitis, initially attributed to interferon‐induced drug reaction, which led to therapy discontinuation. Treatment with cyclosporine (200 mg/day) and methotrexate (15 mg/wk) was started, aiming at targeting possible pathogenic mechanisms underlying granulomatous lesions. By 2023, the patient experienced persistent inflammation and ongoing systemic symptoms, such as fever and fatigue, while autoimmune tests remained negative. In the following months, she developed epistaxis and haemoptysis. A high‐resolution computed tomography (CT) scan showed multiple pseudonodular consolidations in her lungs, and a new biopsy confirmed granulomatous inflammation (Figure 1D and E). Given the evolving clinical picture, ANCA testing was repeated, revealing high PR3‐ANCA titer (67 IU/mL; positive threshold > 3 IU/mL). These findings led to a revised diagnosis of mass‐forming GPA.

## Differential diagnosis

The presence of foamy histiocytes was a key diagnostic challenge because these cells can be observed in ECD, Rosai–Dorfman disease, Langerhans cell histiocytosis, Whipple disease, and certain mycobacterial infections.^2^ Extensive microbiological testing was negative, and histopathological review ruled out ECD, leading to an interim diagnosis of “granulomatous disease not otherwise specified.” Re‐evaluation of the initial breast biopsy confirmed chronic inflammation with necrotizing granulomas and foamy histiocytes. Additional histochemical analysis excluded Langerhans cell histiocytosis (negative CD1a and S100 staining) and malakoplakia (absence of Michaelis–Gutmann bodies). Given persistent systemic inflammation, emerging pulmonary nodules, and ear–nose–throat involvement, repeat ANCA testing confirmed PR3‐ANCA positivity, solidifying the diagnosis of GPA.

## Management and outcome

Rituximab induction therapy (two doses of 1 g each, two weeks apart) was initiated, followed by maintenance per MAINRITSAN protocol.^3^ Cyclosporine was discontinued, methotrexate was continued, and steroids were tapered off within a year. The patient achieved clinical remission with sustained resolution of symptoms, declining ANCA titer and normalization of markers of inflammation at the last follow‐up in 2024.

## Discussion

This case illustrates the diagnostic complexity of mass‐forming GPA, an entity scarcely reported in the literature. The presence of foamy histiocytes initially misled the diagnosis toward ECD because these cells are rarely described in AAV. Only a single report in literature associates foamy histiocytes with GPA.^4^ The atypical multiorgan presentation, including breast and thyroid involvement, further delayed diagnosis. Mass‐forming GPA is often mistaken for malignancies or infections, and the absence of ANCA at disease onset complicates recognition. Literature suggests that these cases require aggressive immunosuppressive therapy, including glucocorticoids, rituximab, or cyclophosphamide.^5^

## Conclusions

Mass‐forming GPA is a rare, underrecognized entity that often leads to diagnostic delays. This case underscores the challenges posed by atypical histopathological findings, such as foamy histiocytes, and highlights the necessity of repeated ANCA testing in unexplained granulomatous diseases. Rituximab was effective in achieving remission, reinforcing its role in managing this rare GPA phenotype.

## AUTHOR CONTRIBUTIONS

All authors contributed to at least one of the following manuscript preparation roles: conceptualization AND/OR methodology, software, investigation, formal analysis, data curation, visualization, and validation AND drafting or reviewing/editing the final draft. As corresponding author, Dr Quartuccio confirms that all authors have provided the final approval of the version to be published, and takes responsibility for the affirmations regarding article submission (eg, not under consideration by another journal), the integrity of the data presented, and the statements regarding compliance with institutional review board/Declaration of Helsinki requirements.

REFERENCES1

Quartuccio
L
, 
Treppo
E
, 
Urso
L
, et al. Unmet needs in ANCA‐associated vasculitis: physicians’ and patients’ perspectives. Front Immunol
2023;14:1112899.36911748
10.3389/fimmu.2023.1112899PMC99953792

Shah
KK
, 
Pritt
BS
, 
Alexander
MP
. Histopathologic review of granulomatous inflammation. J Clin Tuberc Other Mycobact Dis
2017;7:1–12.31723695
10.1016/j.jctube.2017.02.001PMC68502663

Tanna
A
, 
Pusey
C
. Clinical trials. Rituximab for maintenance of remission in AAV. Nat Rev Nephrol
2015;11:131–132.25599619
10.1038/nrneph.2014.2544

Keorochana
N
, 
Klanarongran
K
, 
Satayasoontorn
K
, et al. Necrobiotic xanthogranuloma scleritis in a case of granulomatosis with polyangiitis (Wegener's granulomatosis). Int Med Case Rep J
2017;10:323–328.29042820
10.2147/IMCRJ.S145943PMC56332935

Padoan
R
, 
Campaniello
D
, 
Gatto
M
, et al. Current clinical and therapeutic approach to tumour‐like mass lesions in granulomatosis with polyangiitis. Autoimmun Rev
2022;21:103018.34902605
10.1016/j.autrev.2021.103018

## Supporting information


**Disclosure form**.
